# Subcutaneous C1‐Inhibitor Concentrate for prophylaxis during pregnancy and lactation in a patient with C1‐INH‐HAE

**DOI:** 10.1002/ccr3.3743

**Published:** 2021-02-06

**Authors:** Shimalee Andarawewa, Emel Aygören‐Pürsün

**Affiliations:** ^1^ Hereditary Angioedema Center Department of Children and Adolescents University Hospital Goethe University Frankfurt am Main Frankfurt Germany

**Keywords:** C1‐inhibitor concentrate, hereditary angioedema, lactation, pregnancy, prophylaxis

## Abstract

Subcutaneous plasma‐derived human C1‐Inhibitor concentrate (pdC1INH) may be safe and effective for long‐term prophylaxis during pregnancy and lactation in hereditary angioedema patients.

## Graphical abstract

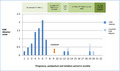

## 1 INTRODUCTION

Hereditary angioedema (HAE) is an autosomal dominant disease caused by a deficiency of C1‐Inhibitor and is clinically manifested by recurrent episodes of localized subcutaneous or submucosal edema lasting for 2‐5 days. It is a clinical challenge to manage HAE patients during pregnancy due to potential worsening of the disease with the physiological increase in estrogens.^1^, ^2^ In about 60% of pregnant patients with HAE, an increased frequency of attacks during pregnancy has been shown and in about 40% of these patients symptoms were seen throughout the pregnancy.^3^ Added to that, there are limited treatment options during pregnancy.^1^, ^2^ These comprise of on demand intravenous (iv) plasma‐derived human C1‐Inhibitor concentrate (pdC1INH) for acute attacks and iv pdC1INH or Tranexamic acid per os for prophylaxis.^2^, ^4^

## 2 CASE HISTORY

A known hereditary angioedema (C1‐INH‐HAE Type 1) female patient, age 38 years, who had a mild course of disease, started experiencing significant increase in angioedema attacks during her first pregnancy. The patient was diagnosed as having HAE at the age of 17 years when she had her first swelling. When she started taking the contraceptive pill the frequency of attacks increased. Cessation of the estrogen contraceptive pill improved her condition and thereafter she was prescribed a progesterone only pill. Since then the patient had a mild course of disease with the number of attacks per year ranging from 0 to 2. The attacks were treated with 500‐1000 units (U) C1‐INH iv by the general practitioner.

When the progesterone only pill was stopped due to child planning, the patient had 4 swellings in the extremities per year. During the first 2 months of the pregnancy, the patient had 2‐3 attacks per month. The patient treated only severe attacks, so the untreated swellings lasted longer. She often had episodes of new swellings daily, as well as multiple swellings in different parts of the body at the same time, with considerably reduced quality of life. Added to that, a laryngeal edema attack caused hospitalization of the patient. She continued working full‐time. The patient was feeling uneasy and feared HAE attacks and later on developed depression symptoms. The patient and her husband were trained to inject C1‐Inhibitor concentrate intravenously in the meantime and advised to treat attacks as soon as possible.

The frequency of attacks remained twice a week at the end of the second trimester, which was a great burden to the patient. Poor venous access complicated the iv therapy of HAE attacks. We started subcutaneous (s.c.) pdC1INH prophylaxis at this stage with 1500 U twice a week. The patient was trained to inject subcutaneous medication at our center. Under this treatment, she had no swellings from the beginning of subcutaneous prophylaxis until delivery.

The patient had a spontaneous vaginal delivery in the 38th gestational week and gave birth to a healthy child. The subcutaneous prophylaxis was continued to cover the postpartum period. The patient started taking the progesterone contraceptive pill approximately 1 month after the delivery.

**FIGURE 1:**
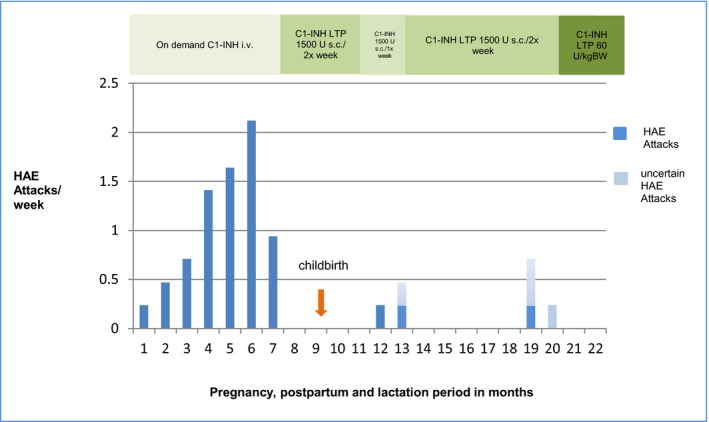
Course of HAE‐attack frequency during pregnancy and postpartum period of the index patient *(HAE Attacks per week = Attacks per Month divided by 4.25 to receive attacks per week)*

About 7‐week postpartum, as the patient was free of attacks, the subcutaneous pdC1INH prophylaxis was to be decreased gradually. The patient attempted on her own to reduce the frequency of injection to once a week 1500 U of pdC1INH s.c. for 4 weeks. When she stopped the injection for 8 days she had a foot swelling so she continued with 1500 U of pdC1INH s.c. once a week for further 3 weeks. She then had another foot and elbow swelling. Three days afterward she had a lump in the throat feeling and was treated with on demand pdC1INH. Thereafter, she continued on a twice a week 1500 U of pdC1INH s.c. prophylaxis regime and remained without swellings until almost 10 months after childbirth (Figure 1). Ten Months after childbirth, due to increased stress, the patient had a uvula swelling with laryngeal edema symptoms, which was treated with pdC1INH iv in the hospital. Thereafter, the patient had recurrent symptoms suggestive of laryngeal edema with a lump in the throat feeling (without hoarseness of voice, dysphagia or dyspnea) three to four times within the next 2‐3 weeks and she treated herself with pdC1INH iv accordingly. Due to the critical site of potential angioedema, the prophylactic dose was increased to the recommended dose of 60 U/kg body weight pdC1INH s.c. twice weekly, under which the patient remained attack free up to date.

## 3 DISCUSSION

In the case presented, subcutaneous pdC1INH was a successful and safe treatment for prophylaxis in HAE during pregnancy and lactation. This patient was relieved and fully satisfied with the above prophylaxis. Subcutaneous pdC1INH prophylaxis during pregnancy was not a recognized treatment option at the time this patient was treated, but has been approved in some countries recently. However, during pregnancy, other available subcutaneous medication for HAE is not recommended. Therefore, subcutaneous pdC1INH prophylaxis maybe a valuable treatment option in these patients. To our knowledge, this is one of the few cases ever reported with subcutaneous pdC1INH prophylaxis during pregnancy and breastfeeding. Mumneh et al reported 2 cases treated with subcutaneous C1‐INH prophylaxis during pregnancy with good efficacy and safety. As in our case, there were no reported congenital abnormalities.^5^ Levy et al described four pregnant women with exposure to pdC1INH 40‐60 U/kg prophylaxis for four to 8 weeks during the COMPACT trial (Clinical Study for Optimal Management of Preventing Angioedema with Low‐Volume Subcutaneous C1‐Inhibitor Replacement Therapy) with a favorable pregnancy outcome.^6^

## 4 CONCLUSION

Our present case indicates that pdC1INH s.c. is a successful and safe treatment option for prophylaxis in C1‐INH‐HAE during pregnancy and lactation and may be effective in less than recommended doses over long periods of time. This case also adds to the clinical evidence of safety and efficacy of subcutaneous C1‐INH for prophylaxis in HAE.^5^

## ACKNOWLEDGMENTS

Open access funding enabled and organized by ProjektDEAL.

## CONFLICT OF INTEREST

SA reports nonfinancial support from CSL Behring, personal fees and nonfinancial support from Takeda/Shire. EAP reports grants and personal fees from Biocryst, grants and personal fees from CSL Behring, grants and personal fees from Kalvista, personal fees from Pharming, grants and personal fees from Shire/Takeda.

## AUTHOR CONTRIBUTIONS

SA and EAP: contributed to acquisition of clinical data and wrote (SA) and reviewed the manuscript critically (EAP).

## ETHICAL APPROVAL

Ethics review exempted.

## CONSENT

Written informed consent was obtained from the patient for publication of this case report.

## Data Availability

The data reported are available from the corresponding author on reasonable request.
